# Implementation Considerations for Family-Based Telehealth Interventions for Youth in Foster Care: Focus Group Study With Child Welfare System Professionals

**DOI:** 10.2196/45905

**Published:** 2023-12-29

**Authors:** Hannah P Leo, Johanna B Folk, Christopher Rodriguez, Marina Tolou-Shams

**Affiliations:** 1 Department of Psychiatry and Behavioral Sciences University of California, San Francisco San Francisco, CA United States; 2 Child and Adolescent Psychiatry New York-Presbyterian New York, NY United States

**Keywords:** foster youth, telehealth, family-based interventions, mental health

## Abstract

**Background:**

Between 2016 and 2020, over 600,000 youth were served annually by the foster care system. Despite approximately half of foster youth struggling with emotional or behavioral challenges, few receive much-needed services to address their mental health concerns. Family-based interventions are efficacious in addressing both youth and caregiver mental health needs; however, foster youth participation in these family-based interventions is limited by many barriers, including out-of-home placement far from their family of origin. Telehealth is a promising tool for mitigating barriers to access to treatment interventions for foster youth and their families.

**Objective:**

This study aims to understand child welfare system professionals’ perspectives on enabling factors and barriers to providing family-based interventions via telehealth to youth in out-of-county foster care placement.

**Methods:**

This qualitative study derived themes from 3 semistructured focus groups with child welfare system professionals. Participants were asked to discuss how family-based interventions are delivered to foster youth and their caregivers in their jurisdictions, as well as to share their thoughts about how to use telehealth to improve access to family-based interventions for families with youth in out-of-home placement. Data were analyzed using constant comparative analysis and inductive thematic analysis, with the Behavioral Model for Vulnerable Populations as the theoretical framework.

**Results:**

Participants were 19 child welfare system professionals (eg, social workers, residential treatment staff, and supervisors) who participated in 1 of the 3 focus groups (6-7/group). Most participants were women (n=13, 68%), White individuals (n=10, 53%), and social workers (n=8, 42%). On average, participants worked in the child welfare system for 16.6 (SD 8.3) years. Participants identified multilevel factors impacting family-based intervention delivery including environmental factors (eg, Medicare billing and presumptive transfer), predisposing characteristics (eg, psychological resources), enabling factors (eg, transportation and team-based youth-centered care), and need factors (eg, motivation to engage). Participants expressed optimism that telehealth could increase access to needed mental health care, diverse providers, and longevity of care while also expressing some concerns regarding telehealth access and literacy.

**Conclusions:**

Child welfare system professionals highlight the need to develop policies and telehealth interventions that are youth versus placement centered, include resources that limit barriers and bolster motivation for engagement, and follow a team-based care model. Findings from this study inform how telehealth can be used to increase access to and engagement with family-based interventions for youth in out-of-home placements and their caregivers of origin.

## Introduction

### Background

Approximately one-third of all children and over half of Black children in the United States experience a Child Protective Services investigation for maltreatment (ie, physical, sexual, or emotional abuse or neglect) before the age of 18 years [1]. In 2020, a total of 631,832 youth were served by foster care (ie, out-of-home placement organized by child welfare services) [2], with Black and Indigenous youth having the highest risk of placement [3]. Although nearly half of foster youth exhibit clinically significant emotional and behavioral needs, one study found that only 11.7% connected with mental health services within 1 year of maltreatment investigation. Access is especially limited for Black and Latinx foster youth [4,5].

Family-based interventions are the gold standard for addressing externalizing and internalizing mental health disorders in adolescents, including foster youth [6,7]. Strengthening interpersonal relationships between youth and caregivers bolsters a youth’s social competence and behavioral adjustment, reduces caregiver stress, and supports family reunification [7,8]. Unfortunately, many foster youth are placed far from caregivers (eg, in January 2022, in Northern California Bay Area counties, 22%-66% of youth aged 0-21 years were placed out of county) [9], making in-person family-based interventions challenging.

Telehealth, which has been shown to have comparable feasibility, outcomes in assessment and symptom reduction, and satisfaction as in-person care, may circumvent this barrier and improve access to mental health interventions [10-12]. Telehealth service delivery for foster youth and families has been shown to maintain service quality, improve access and engagement, and be perceived by users as beneficial [13-15]. Recently, there has been increased interest in telehealth, as the COVID-19 pandemic has provided the impetus for the rapid adoption of videoconferencing in many settings, including the juvenile justice system, which also separates youth from their families. Given the feasibility and acceptability of telehealth service delivery, there has been a call to leverage technology for mental health interventions for system-involved youth and their families [16]. While research into telehealth delivery of mental health services is gaining momentum, little is known about the effectiveness of family-based telehealth interventions for foster youth and their families or how best to implement such services.

### Objective

The aim of this study was to understand child welfare system professionals’ perspectives to inform the adaptation of an existing empirically supported, in-person, family-based affect management intervention for telehealth delivery tailored for foster youth.

## Methods

### Study Design

This study analyzes focus group data to guide the iterative adaptation of an empirically supported, in-person, family-based affect management intervention [17] to serve foster youth and their families via telehealth as part of phase 1 of the Family Telehealth Project. The overall study aims to improve behavioral health outcomes and reduce housing instability among adolescent foster youth (aged 12-18 y) placed out of county, for whom in-person family-based interventions with caregivers of origin are typically not feasible [18]. Phase 2 is an ongoing clinical trial to evaluate the effectiveness of this intervention.

### Sample

Eligible participants were English-speaking child welfare system professionals (eg, social workers, supervisors, and program staff) working with foster youth and their families. Staff at child welfare services in a Northern California county and a short-term residential therapeutic program (STRTP) in Southern California, a placement for some Northern California youth, were recruited by phone and email. Eligibility screening was performed and informed consent was obtained over phone (using DocuSign to document written consent) or in person before the focus group. Eligible individuals were invited to a one-time focus group at their workplace. Additional participants were identified by snowball sampling.

### Data Collection

Three in-person 90-minute focus groups were conducted between December 2019 and February 2020, with 6 to 7 participants per group, a notetaker, and facilitators. Facilitators used a standardized semistructured guide targeting (1) the current landscape of family-based interventions for foster youth, (2) perspectives on content topics for a family-based intervention, and (3) recommendations for telehealth delivery (eg, timing). The participants completed a brief demographic questionnaire at the end of the focus group. Focus group discussions were audio recorded, transcribed verbatim, and deidentified. Facilitators and notetakers used written notes to draft debriefs including key themes and issues to address in the next focus group (eg, unclear questions and timing).

### Ethical Considerations

All study procedures were approved by the institutional review board of the University of California, San Francisco (number 19-28922). The focus group participants provided written informed consent before participation. Personal data collected in this study included information about the system professionals’ demographics, thoughts about telehealth and family-based interventions for child welfare–involved families, and digital recordings of focus groups. No names or other identifying information was included in the analytic data and during focus groups. Participants were reminded frequently not to indicate their name or any identifying information about themselves or others. Focus group transcripts were deidentified before analysis. Participants were provided with food during the focus group and, if allowed by their agency, offered a US $40 Visa gift card as compensation.

### Qualitative Analysis

Using inductive thematic analysis, 3 authors reviewed transcripts and created an initial coding scheme [19]. Two authors independently coded all focus group transcripts, updated the coding scheme to resolve coding differences, reviewed each transcript twice to create a master coding scheme, and reviewed again to develop master transcripts coded in ATLAS.ti (version 8.0; ATLAS.ti Scientific Software Development GmbH) [20] for each focus group. Two coders drafted and revised analytic memos using the constant comparative analysis method [21] to identify and categorize key themes from the data [22].

### Theoretical Framework

The Behavioral Model for Vulnerable Populations [23] represents a revision of the Behavioral Model of Health Services Use [24] and was used as a theoretical framework to organize emerging themes from the focus groups. This model suggests that health care utilization is influenced by a population’s predisposing characteristics, enabling resources, and overall need [24]. Subsequent revisions to the original theoretical model incorporated environmental factors (health care system and external environment), health outcomes, definitions of health behavior as personal health practices and health service utilization, and feedback loops to emphasize interactions between components [24]. The Behavioral Model for Vulnerable Populations is an adaptation designed to understand health services utilization among underserved populations including people from minoritized groups, children, and those with housing instability by adding “Vulnerable Domains,” in addition to “Traditional Domains,” for each variable (eg, mental illness in predisposing characteristics; competing needs and transportation in enabling factors) [23]. With “Vulnerable Domains” variables in mind, our data are presented using a version of the model published by Phillips et al [25], highlighting contextual factors including provider-related variables. The authors acknowledge that the term “vulnerable” is problematic given the placement of blame on the individual rather than on an inadequate system; however, the term is used for consistency with the theoretical model [23].

## Results

### Overview

Participants (N=19; 6-7/group) predominantly identified as women (n=13, 68%), White (n=10, 53%), non-Latinx (n=14, 74%), and social workers (n=8, 42%). On average, participants worked in child welfare services for 16.6 (SD 8.3) years. Table 1 presents additional demographic information.

**Figure 1:**
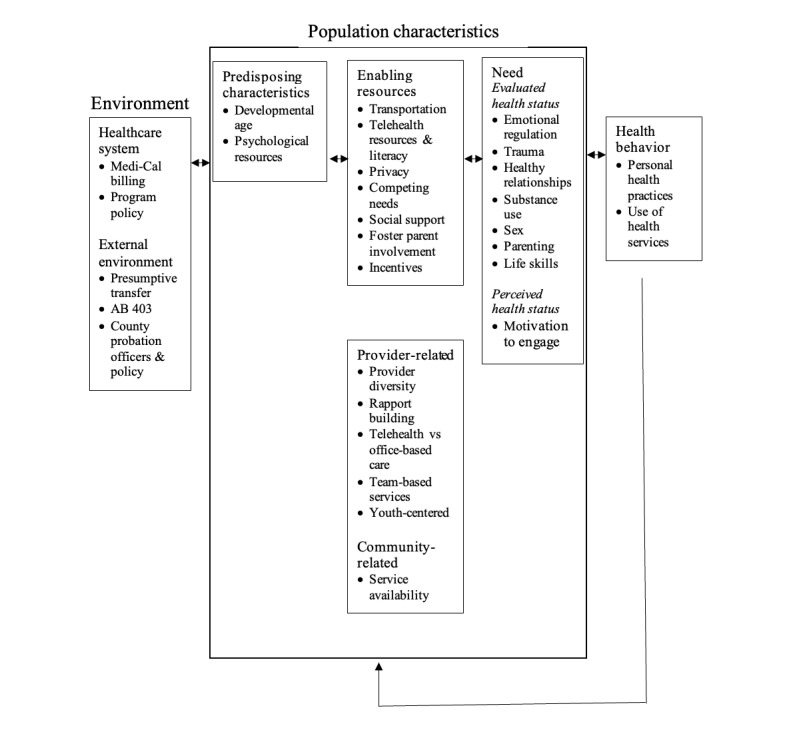
Factors, per child welfare system professionals, influencing the implementation of family-based telehealth interventions for foster youth and their families. Organized using the Behavioral Model of Health Services for Vulnerable Populations [23] and Utilization Model [25], both adaptations of the Behavioral Model of Health Services Use [24]. AB: Assembly Bill.

Participants identified multilevel factors impacting family-based intervention utilization (Figure 1), including environmental factors (Table 2) and population characteristics (Table 3).

**Table 1:** Demographic characteristics of child welfare system professionals who participated in 1 of 3 focus groups (N=19).

Characteristic				Participants, n (%)
**Gender**				
	Women		13 (68)	
	Men		6 (32)	
**Age (y)**				
	25-34		5 (26)	
	35-44		7 (37)	
	45-54		5 (26)	
	55-64		2 (11)	
**Race^a^**				
	American Indian or Alaska Native		1 (5)	
	Asian		2 (11)	
	Black, African American, or Haitian		4 (21)	
	White		10 (53)	
	Mixed race		2 (11)	
	Other^b^		4 (21)	
**Ethnicity**				
	Hispanic or Latinx		5 (26)	
**Setting and role**				
	**Human services agency**		12 (63)	
		Case manager	1 (5)	
		Social worker	8 (42)	
		Supervisor	3 (16)	
	**STRTP^c^**		7 (37)	
		Mental health or substance use coordinator	2 (11)	
		Psychotherapist	1 (5)	
		STRTP staff	2 (11)	
		Administrator	1 (5)	
		Supervisor	1 (5)	

^a^Categories are not mutually exclusive and reflect how participants self-identified.

^b^Other: Three wrote Latinx or Hispanic. One wrote Mexican/French.

^c^STRTP: short-term residential therapeutic program.

**Table 2:** Environmental variables affecting the utilization of family-based interventions for foster youth and families per child welfare system professionals.

Variable		Example quotations
**Healthcare system variables**		
	Medi-Cal billing	“One thing, I just was curious about how it would relate with other services being provided. In other words, if there would be a limitation on other therapy. Because, you know, Medi-Cal only allows for one person to be billing for individual therapy with a given client, so I just don’t know if it would be a Medi-Cal billing thing or funded some other way. I don’t know.” [Social work supervisor]
	Program policy	“Sometimes we’ll do an exception, but it’s hard to get those... Like if it’s 97 miles, they’ll do an exception. That’s what I mean, not an exception based on what the youth’s needs, as much. It’s really, I mean, definitely, I think that is taken into account, like if the kid is really attached, but I’ve never seen, correct me if I’m wrong, an exception for 150 miles or something.” [Social worker]
**External environment variables**		
	Presumptive transfer	“And it definitely causes a lot of barriers, because we have some kids where we try to do split services where they have some providers in their county, but let’s just say they’ve had their psychiatrist for five years and it is drivable. The foster parent is like, ‘Yes, once a month, I have no problem. I will drive an hour so this kid can keep their psychiatrist, who’s been working really well with them.’ But now we have a billing issue... So, it also impacts keeping that consistency... so it kind of plays back into that the services follow the placement versus the youth.” [Supervisor]
	Assembly Bill 403	“If you go to one STRTP^a^, they get the treatment there at that STRTP. And then if they move to a different STRTP, their treatment providers are going to end and be over here at this STRTP. And if I’m a kid that’s in a foster home and I have a wraparound team, then I move into a STRTP for some reason, I’m going to lose my wraparound team and I’m going to have to accept the treatment through the STRTP. So, for me, this is one of those laws where it seems that the state was trying to help, and it’s not at all clear to me that they did.” [Supervisor]
	County POs^b^ and policy	“And some POs don’t let kids go home on home visits to test out and practice the skills they’re working on. Like, home visits are not just like, ‘Hey, I’m going home to chill and party and do whatever.’ The intended purpose of home visits are to practice the skills you’re learning in placement. We have things where there’s family agreements and different things where they’re learning new skills, how we’re going to problem solve, what are we going to do to take a time out? What are we doing in terms of communication when we have conflict? How are parents able to reinforce boundaries and natural consequences? All those things, that’s what we’re trying to teach in our program and accountability, and they just don’t get to practice that without having a home visit. I have very strong feelings about this.” [Administrator]

^a^STRP: short-term residential therapeutic program.

^b^PO: probation officer.

**Table 3:** Population characteristics affecting the utilization of family-based interventions for foster youth and families, per child welfare system professionals.

Variable				Example quotations
**Predisposing variables**				
	Developmental age			Regarding the transitional age period being especially vulnerable: “It is, and a lot of them, because of the trauma they’ve experienced, their biological age is not necessarily matching their emotional, developmental [age].” [Social worker]
	Psychological resources			“...They have this history of all these things that happened, and it just feels overwhelming and didn’t go anywhere. Working on specific skills and things, that seems easier to master.” [Social worker]
**Enabling resource variables**				
	Transportation			“It is a trek. It is a bus, it is a BART^a^, then another bus, and then a taxi, and then a this. And we have some parents that will do it religiously. We have other parents that, it’s rough, it’s a rough trek and they’re not able to do it as much.” [Supervisor]
	Telehealth resources and literacy			“Then it raises the questions of access to technology and access to the internet service that would, no doubt, be needed. And you know, the technology itself then becoming an obstacle, also...” [Supervisor]
	Privacy			“I’m less comfortable with people in the same room where I can kick her, and I can tell her different things. I am a little concerned, but not overly so, of the confidentiality of I have someone else listening in who is then going to call up my kid after we get off the phone and berate them for whatever was said, but that could happen anywhere.” [Supervisor] “It could be difficult for bio parents if they don’t have a private space with Wi-Fi. Like if they’re used to going to the library for Wi-Fi or café for Wi-Fi and you need a private space with confidentiality, like quiet, if they’re living in an SRO^b^, something like that, it could be more difficult.” [Social worker]
	Competing needs			“For this population, just scheduling, they all, they work on the weekends.” [Psychotherapist] “...They would rather just be out in the community, hanging out with each other. They’re not going to want to sit in a therapy session during the three times a year that mom can come and visit in-person.” [Social worker]
	Social support			“The first thing that came to my mind was helping the young person by having their parent figure involved in their life, helping them feel more sense of connection and permanency. Because what we’re finding is that when our kids have permanent people in their life, they know that person loves them, no matter if they’re there, touching them, or just their person, they tend to do better in outcomes of getting out of foster care, aging out of foster care.” [Case manager] “It benefits them to know that their mom and dad or their aunt or uncle or cousin, whoever that person is, is there, because that’s the person they go to, not us... That’s the person who they talk to, the person who they ask advice to, and when they leave the system, that’s the person who they’re going to count on, other than us.” [Case manager]
	Foster parent involvement			“...I also have some foster parents who are very experienced, and so they do the visits with the mom at their house and actually show them, ‘Oh, no, this child needs this at this time.’ They show them how to talk to them. Say two children are arguing, then the foster parent would model how the mom should handle the argument, like ask each child what the problem is. I have one foster parent who does great at that, and the mom gets their kids back very soon because they’re working with this foster parent.” [Social worker]
	Incentives			“In my experience, trying to get parents to do something consistently would be a huge barrier...” [STRTP^c^ staff]. “Unless they were getting something out of it, then they might” [Supervisor]. “Yeah, if its incentive based for the parents, then absolutely. Everything, nowadays, seems it has to be incentive based.” [STRTP staff]
**Enabling provider-related variables**				
	Provider diversity			“...I really want to emphasize that having more providers of color would be particularly helpful for a number of our youth.” [Supervisor] “So, if I’m in [one city] and there are no therapists that have history working with LGBT^d^ folks, wouldn’t it be nice for a kid or family or caregiver to be able to connect? And even if that’s not their main source of treatment, but at least to have that person that reflects them whether it looks like them or just reflects them, in general, if it opens up the ability to access more diverse therapists, especially when you’re not in the immediate Bay Area, I think that is a big positive.” [Supervisor]
	Rapport building			“...It’s kind of more of a synergy that’s created and more camaraderie where all of us, collectively, form relationships where the changes happen over a period of time, consistent interaction, and there’s a level of trust for us, as a staff, and a level of trust for them. And the kids see that teamwork, as well...” [STRTP staff] “...And I think that’s key in how we do engagement, like for families to understand we’re not just another piece of the system, but we’re really here to help them...” [Administrator]
	Telehealth vs office-based care			“I think it’s great. Teens are attached to their phones. Some parents are attached to their phones. And they’re on it all day, anyway, and I wonder if there would be a higher level of engagement if the therapy was right on their phone. So, like while they’re checking Instagram, they also have access to that support.” [Supervisor] “And I think there’s certain positives to that screen where, for those hard topics for the youth, like the sexual health, reproductive rights, it’s a little bit safer. It’s kind of like how we have youth who do great in that group home setting, but you put them in a family setting, it all falls apart because it’s too intimate. So, for youth who maybe struggle with that intimacy, it sort of puts that barrier to where they’re talking to someone, but they don’t have to really have that intimate, person-to-person contact.” [Supervisor]
	Team-based services			“ISFC^e^ teams and wraparound teams are like a mini team. They usually have two or three providers that are all assigned to the same youth, doing different things. I think those modalities, generally speaking, are good.” [Supervisor]
	Youth centered			“Because it seems to make more sense that you make the services youth-based, not placement-based, and we are a very placement-based services, when, if the youth get to keep their team, build their team, build the trust, do the hard work because of the trauma, I think we would see more successes. Versus each placement having their own team. It just makes more sense for the teams to follow the youth, because we have some kids that move a lot, and those are obviously the kids with the highest needs, highest mental health, and so we can’t even get a team in place before they have to move again.” [Supervisor]
**Enabling community-related variables**				
	Service availability			“...Because we have kids who have severe mental health and some behavior that comes with it, but then we have kids who have behavior issues, not necessarily the severe mental health, but just some mental health and trauma, and they’re all in the same placements. So, when the kids hit 18, we can start accessing the adult mental health system... but before that, unless they’re a regional center^f^, which is different, it’s just a mixed bag in with all the other youth in the group homes, in the STRTPs, and sometimes, that can be a bit exploitive for youth who aren’t able to kind of stand up to people or really not even know what’s going on. And they can definitely be victimized or bullied or asked to do things, and all of a sudden, they’re getting arrested for carrying around alcohol. And you’re like, ‘Why were you carrying around alcohol?’ Because the other youths told them to carry it.” [Supervisor] “Where we don’t have access to a lot of different modalities. Some of our kids would do great with drama therapy, even still art therapy, drawing, DBT^g^, different modalities for different diagnoses, and we just throw the run of the mill, we’re going to put you in therapy and don’t really match the youth and their needs and their diagnoses to, I guess, an evidence-based modality that has shown to work.” [Social worker]
**Need variables**				
	**Evaluated health status**			
		Emotional regulation, trauma, healthy relationships	“Well, I think, also, there could be a parallel in their emotional regulation. My youths are having trouble day to day, sometimes, regulating how they react to the world, where their parents are probably having a parallel process. So, somehow, kind of going off what you were saying, working with them on small steps on how to, okay, what happens when you get upset?” [Case manager] “I think another topic, if able, to touch on healing. Because I know that, like, detaining or removal, there’s a lot of trauma that came with that from both peoples’ perspective, the parent and the child, and not talking about that, and the parent is working on their case plan or whatever to be ready to have the kid reunified, but that kid has feelings, too. And especially if they’re a teen, probably a lot of feelings. And without that kind of healing work, putting them back into the same place and expecting everything to be fine and moving forward...” [Social worker]	
		Substance use, parenting, sex, life skills	“I would say 80% of the youth that I work with use substances, so that’s definitely at the top there, and managing feelings.” [Social worker] “Especially marijuana, because since it’s legal here, they think it’s okay. They think it’s not bad when it is, for kids.” [Social worker] “One of the things that I would like to see is kind of like pre-counseling, working through some of the living together challenges if they’re going to be potentially moving, because when you have a weekend visit or even a two-week visit, it’s all exciting, you make it lots of fun, but there’s no workup towards what’s everyday life going to look like? What are the rules going to be? Because everybody’s focused on, it’s so exciting, we’re going to go to this restaurant or whatever, I’m going to make your favorite food. So, that would be one thing.” [Supervisor]	
	**Perceived health status**			
		Motivation to engage	“...Willingness to be involved, where they’re multi-stressed families with lots of other demands, and sometimes, as much as they may or may not love their children, it’s one less thing they have to worry about when their kid is in care. I don’t know if that’s true for all families, but I think that can happen. And so, like, it almost feels like sometimes, out of sight, out of mind...” [Administrator] “Give them back when you fix them.” [Supervisor] “Yeah, like, 'It’s your job to take care of this and fix this and we’ll see what we can do when the kid comes back.'” [Administrator] “...A lot of times, the families don’t realize they require some changes and some intervention in terms of their process and approach to whatever the problem that developed.” [STRTP staff]	

^a^BART: Bay Area Rapid Transit.

^b^SRO: single room occupancy.

^c^STRTP: short-term residential therapeutic program.

^d^LGBT: lesbian, gay, bisexual, transgender.

^e^ISFC: intensive supportive foster care.

^f^Regional center: Locally based nonprofit private corporation coordinated by the Department of Developmental Services in California to serve as a local resource to connect individuals with developmental disabilities and their families to services.

^g^DBT: dialectical behavior therapy.

### Environmental Factors

Medi-Cal, state, and program policies were cited by participants as environmental barriers to the continuity of care with mental health providers. Multiple participants described presumptive transfer (policy Assembly Bill 1299: “responsibility for providing or arranging for specialty mental health services shall promptly transfer from the county of original jurisdiction to the county in which the foster child resides”) [26] as a major impediment, given that youth must change providers when moving counties for placement. One social worker explained that while waiving presumptive transfer is an option, obtaining approval often delays services. A social work supervisor wished that Medi-Cal coverage for mental health care would be state-wide rather than county-based coverage to promote consistency and access to providers. Participants also noted the impact on provider continuity of Assembly Bill 403, a policy outlawing group homes (facilities providing 24-hour nonmedical care and supervision to youth aged <19 years who are under court jurisdiction or who are dependents of the court) and instead requiring STRTPs [27]. STRTPs are similar to group homes but are additionally mandated to provide in-house specialized therapeutic treatment [28]. Every time foster youth enter an STRTP, they must establish care with that STRTP’s in-house providers and discontinue care with prior providers.

Program policies limiting the distance a provider can travel to work with youth was cited as a barrier to treatment**.** Participants explained that one local provider of wraparound services, a team-based service delivery model using a strength-based need-driven approach [29], can only travel up to 90 miles, disrupting care when youth are placed farther away. Rare exceptions are made, often based on distance, rather than a youth’s needs.

An administrator cited a county’s Medi-Cal approval process to use telehealth as a barrier; the process involves documenting nuanced policies and procedures that are Health Insurance Portability and Accountability Act (HIPAA) compliant, approval by the mental health contract and the specific mental health program, and an annual review. A social work supervisor also expressed concerns that if the youth are already in individual therapy, additional family-based therapy may not be paid for by Medi-Cal.

Varying roles and policies of probation officers by county were discussed as impacting treatment for youth who are dually involved in juvenile justice and foster care. Participants at an STRTP serving dually involved youth explained that probation officers, depending on the county, have the ability to decide when, how, and which support persons could be involved in a youth’s care, timing of family visits or reunification, and placement of youth in independent living programs versus with their primary support person (if not a legal guardian). They also shared that probation officers may not provide a projected court date for completion of services and possible reunification, which per an administrator, “creates unnecessary anxiety” for youth and limits the ability to use home visits to practice problem solving, communication, boundaries and accountability skills learned in placement.

### Population Characteristics: Predisposing Characteristics

Participants highlighted that a youth’s trauma history can affect their emotion regulation skills, psychological resources, and relationships with caregivers. Multiple participants expressed a desire for family-based interventions to be offered to transitional-age youth. One social worker highlighted transitional age (16-24 years) as an especially vulnerable period:

...And a lot of them, because of the trauma they experienced, their biological age is not necessarily matching their emotional, developmental [age]...

Social worker

In 2 focus groups, participants expressed concerns that youth would feel uncomfortable sharing sensitive information (eg, sexual health) during family-based telehealth sessions with caregivers of origin with whom they are building a relationship or with multiple adults, including providers and family, at once. Two participants commented that frequent meetings with many providers, such as in wraparound services, can be overwhelming for youth. A STRTP staff member added “...and a lot of times, they just want to be kids. Yeah, they just want their downtime, so it really depends on the individual.” Similarly, participants indicated caregiver mental health and lack of openness during sessions as barriers to treatment. A social worker added that it is advantageous when a caregiver has a therapist to support communication between the caregiver’s therapist and the youth’s providers. One social worker explained that both caregivers and youth can be resistant to therapy due to discomfort and believed that a skills-based intervention would be less overwhelming. In contrast, a mental health or substance use coordinator believed that a structured skills-based intervention could be challenging for youth to “stay focused” if they want to discuss other topics with family.

### Population Characteristics: Enabling Resources

A lack of resources (transportation, time, and finances) was highlighted as a barrier to in-person family-based interventions. All 3 focus groups cited transportation barriers, including lack of access to a vehicle, driver, or funding, and difficulty with limited complex public transportation routes. Participants observed that offering transportation resources to families facilitated connections with providers; however, they noted limitations due to liability when directly transporting youth. Limited time and competing needs were highlighted as salient barriers. An administrator described families as “multi-stressed,” and a psychotherapist noted that caregivers’ work caused scheduling constraints. The participants explained that with limited time to be together, many families prefer quality time over therapy. Participants at an STRTP explained that youth at their facility also have competing program and community obligations including school, extracurricular activities, employment, and groups. Some participants believed that providing incentives to caregivers would help increase their engagement in family-based interventions. They reflected on the challenges of consistent caregiver involvement and their experience of seeing incentives work.

In discussions about telehealth, access to resources (technology, technology literacy, privacy) and feasibility were considered. Some participants said that most youth and adults are “attached” to their phones, making engagement and feasibility easier. One social worker commented that many youth without cellular service connected to caregivers via Wi-Fi using WhatsApp or Messenger. Participants expressed concerns that some families have limited access to technology, especially computers and Wi-Fi; a supervisor suggested that the provision of stipends for smart technology or the internet could facilitate participation. Participants discussed programs that provide clients with free smart phones, noting the variable reliability of devices (iFoster and Obama phones). Multiple participants expressed the need to address the limited technology literacy of some caregivers. Participants also discussed privacy concerns including people intentionally eavesdropping, difficulty finding private spaces with Wi-Fi, and providers needing HIPAA-compliant technology. A case manager stated that for some caregivers, a safe environment outside the home is preferred to facilitate family meetings and provide therapeutic boundaries. Alternatively, a supervisor commented that with the flexibility of telehealth, youth can control the level of confidentiality based on their location during sessions.

Social support was discussed as an enabling resource for family-based interventions. Multiple participants expressed an interest in an inclusive definition of family, identifying siblings, foster parents, or any adult figure beyond just parents who can provide consistent social support and develop strong connections with the youth. A supervisor suggested that sibling participation would allow for continued connection and could address mutual trauma from separation. Favoring a broad definition of social support, a social worker explained the following:

12 to 17-year-olds, the reality for the youth that come to us, they’re not going back to the parent.

Social worker

One social worker expressed that in some instances, involving parents can be counterproductive if they have their own mental health needs and are not ready to engage as caregivers. An additional caveat discussed was “because a lot of youth are unaccompanied, when they come into foster care, the parents’ services get bypassed,” and family therapy is not offered**.** They emphasized consistency and connection, understanding that their work with youth is time limited.

Participants suggested involving foster parents to collaborate with caregivers on consistent rules, model parenting skills during visits, and help with logistics, such as transportation. Participants added that their involvement could be an opportunity for youth to meet foster families before placement. Two social workers explained that increased foster parent involvement could be particularly effective closer to reunification. A social worker explained that court permission must be granted to biological parents to visit the foster homes. An additional barrier, especially for younger youth, is some foster parents’ unwillingness to have biological parents in their homes or know their address.

### Population Characteristics: Provider-Related Variables

Participants identified several provider-related enabling factors for family-based interventions, including rapport with families, team-based services, a youth-centered approach, and choice of service delivery modality. A case manager shared that some families mistrust government agencies, and multiple participants emphasized that building relationships leads to better engagement of families with interventions. An STRTP administrator explained providing “food or furniture or whatever they need to pass a home assessment,” “helping them get into services,” or “see(ing) their kid be engaged” in the program can help build trust. Participants shared the value of “trying our best to empower the family and really establishing the fact that we’re here to work as a team and you’re the expert on your child” in addition to reinforcing “we’re here to help, we’re not here to judge.” A supervisor observed that engaging creatively in youth interests, such as “therapy while shooting baskets... [going] out to lunch, or... therapy while taking a walk” is particularly effective for rapport building.

Two focus groups highlighted team-based models of care that support collaboration among agencies and service providers as important for treatment success. Wraparound services and an adaptation called intensive supportive foster care were explained as successful team-based models of 2 to 3 providers including case managers, care coordinators, therapists, and psychiatrists. Unlike wraparound services, intensive supportive foster care is attached to a particular foster family agency. A participant at an STRTP explained that their program uses a team-based model, including caseworkers who are present as a “source of stability,” care coordinators who serve as mental health clinicians and address acute escalation, and clinical supervisors. Participants explained that having a diverse team of providers facilitates rapid intervention when situations escalate. Additional team members can include the youth, caregivers, youth’s attorney, court appointed special advocate (CASA) mentors (ie, volunteer court–appointed special advocates who often follow youth from placement to placement), and aftercare workers who check in with the family. Participants emphasized the importance of a communication workflow between clinicians delivering a telehealth intervention and the family’s primary clinician in the event of a crisis.

Multiple participants cited the importance of youth- and family-centered services:

I might direct someone to be a little more strategic or structural versus experiential based on what’s going on within a family.

Supervisor

A youth-centered approach is also manifested through “family team meetings where the whole team and the family and the youth get together*.”* As previously discussed, billing by placement is a major barrier to prioritizing needs of youth, who may take time to build trust with their providers. Multiple participants commented that the session frequency, length, and topics covered should be guided by a youth’s needs. Furthermore, some participants agreed with youth requests for diverse providers who share their identity characteristics (ie, sexual identity, race, ethnicity, culture, and gender) to engender trust. Multiple participants commented on the lack of providers who can provide services in non-English languages, resulting in “a lot get[ting] lost in translation.” Participants believed that interventions would be more successful if clinicians had expertise working with diverse populations, including those with low literacy levels, monolingual Spanish speakers, and youth with developmental delays.

When considering the modality for providers to deliver family-based interventions, participants discussed the advantages and disadvantages of telehealth. Multiple participants favored in-person, video, or hybrid sessions over phone-based sessions. Participants expressed that incorporating some in-person sessions could provide “that real element” and have clinical advantages. Many agreed that the engagement and tolerance of session length varies based on the modality and amount of face-to-face interaction: in person>video>phone. The perceived benefits of telehealth included eliminating transportation barriers, consistency of care for youth who moved for placement or college, flexibility of when and where sessions take place, rapid connection to clients during emergencies, access to diverse providers with shared identity, ease of discussing sensitive topics on screen versus in person, and increased engagement due to high phone use among adults and youth. Concerns included youth “clicking away at something else” and thus needing in-person supervision, interventionists missing behavioral cues making de-escalation and rapport building harder, youth feeling intimidated with multiple adults on screen and no support system physically with them, and families having limited exposure to a youth’s progress leading to less engagement in treatment planning, as well as the aforementioned barriers related to access to technology, privacy concerns, and billing and program policies.

### Community-Related Variables

Multiple participants discussed limited appropriate service availability as a barrier to treatment, especially for youth with cognitive delays, dual diagnosis, serious mental illness, behavioral challenges, and/or parenting their own children. Participants explained that they must use general referrals even when the service is unable to meet the youth’s unique needs, and this may expose them to other youth who could exploit them. When programs do exist, they may be unfeasibly distant. In addition, a social worker explained how talk therapy is used for everyone but does not work for many. Even when different therapy modalities are available, the limited quantity of services and associated waitlists are prohibitive. Multiple participants discussed how the quantity and quality of resources varies by county. Smaller counties were noted to have fewer staff and less robust services than counties with better funding and greater monitoring and accountability for services. When services are available, distance and transportation pose barriers to access.

### Need Variables

Participants discussed how variables influencing emotional and behavioral symptoms are areas of evaluated need that could benefit from therapeutic interventions. An STRTP staff member explained that the extent of behavioral challenges affects how much family-based work is needed before home visits or reunification. The suggested session topics included emotional regulation, trauma, fostering healthy relationships, substance use, sex, parenting, and life skills. A social worker explained youth’s complex emotions may manifest as acting out. A case manager observed “parents are probably having a parallel process” on “regulating how they react to the world.” Trauma and the associated emotions especially those related to entry into foster care were suggested topics in all 3 focus groups. Describing the trauma related to entry into foster care, participants explained that many youth are hoping for an apology from their caregivers of origin. Additional suggested topics included domestic violence and healthy relationships, including from whom to seek support from, “how to deal with your feelings about wanting love and how to get it in a healthy way,” and understanding how interpersonal conflict between youth and caregivers manifests in other relationships. A social worker commented that “socioeconomic dependence” in relationships is relevant but overlooked. Substance use, especially marijuana, was perceived as prevalent; one social worker estimated that 80% of the youth with whom they have worked with use substances. A participant at an STRTP serving dually involved youth thought that discussing delinquency or justice involvement would be relevant. Although multiple participants agreed that sexual health is an important topic, some were apprehensive of youth being comfortable with intimate discussions with their caregivers with whom they are building a relationship or with multiple adults. Some worried that dually-involved youth would also be uncomfortable with intimate discussions when probation was involved in the parent-child relationship. They stated that youth may be more comfortable discussing intimacy individually with a noncaregiver. Participants commented that rule setting is challenging for many biological and foster parents. They perceived benefits to setting “ground rules, expectations, and compromises” before placement or before reunification. In addition, parenting as a youth was suggested as a topic for a subset of clients. Independent living skills, including “financial skills, like budgeting, housing”; “organization, like calendar, to-do lists”; “following through, like to appointments”; “returning calls”; and “goal setting” were suggested in all 3 focus groups.

Motivation to engage in care can be viewed as a proxy for perceived health status and self-evaluated need for services. Multiple participants reported a perceived lack of motivation to engage by some youth due to forced therapy with multiple providers. Participants commented that many youth are ambivalent and frustrated with retelling their story to paid professionals. They may also be more willing to engage in therapy, depending on their treatment stage and how interesting they find a therapy session. The perceived lack of motivation of some caregivers to engage or have their youth engage in treatment is also a barrier. The participants cited competing demands, displaced responsibility, and resistance to change as factors that diminish motivation and perceived need. An STRTP staff member explained that many families do not realize that they must play an active role in treatment. Per stakeholders, caregiver motivation to engage can be hindered by limited exposure to youth progress.

## Discussion

### Principal Findings

When developing and implementing an intervention to best serve foster youth and their families, child welfare system professionals’ perspectives are invaluable, given their knowledge of barriers and enabling factors. Participants identified environmental and population characteristic variables, emphasizing the complexity of structural and individual aspects that influence service utilization and treatment. Barriers to family-based telehealth interventions, especially relevant to foster youth and their families, include placement-focused care due to policies restricting the delivery and payment of treatment; perceived lack of engagement; and less access to transportation, technology, privacy, and time. Enabling factors include social support, foster parent involvement, and financial incentives. Providers can further support success by prioritizing youth-centered team-based care, minimizing changes in providers, increasing provider diversity and specialized service availability, and focusing on intervention topics relevant to lived experiences. Notably, these themes overlap with a systems of care framework, which includes the core values of choosing services and supports that are (1) family driven and youth guided, (2) community based, and (3) culturally and linguistically competent [30].

This study supports telehealth as a promising modality to minimize barriers and bolster enabling factors when delivering family-based interventions to foster youth and their caregivers of origin. The study participants were optimistic that telehealth could improve access to consistent care, remove transportation barriers, and better match diverse or specialized providers to youth. Previous studies suggest that foster youth and their families view telehealth as favorable and effective in reducing barriers to engagement, such as transportation [13,14]. A recent study indicated that telehealth service delivery for foster youth is comparable to in-person service delivery, and families attended more appointments via telehealth than in-person services [15]. In addition, although this study occurred before the COVID-19 pandemic, during which telehealth was rapidly implemented, our findings align with those of a survey of foster youth, caregivers, and foster parents during the COVID-19 pandemic; the survey had most participants self-identifying as Black or Latinx that is reflective of the overrepresentation of Black and Latinx youth in child welfare. Of the 228 participants, 77.2% (n=176) found telehealth to be of high quality, and 196 participants indicated telehealth as useful in increasing access and continuity of care [14].

### Strengths and Limitations

A major strength of this study is that multiple perspectives were captured, including those of social workers, STRTP staff, mental health or substance use coordinators, case managers, and supervisors, who are well positioned to identify system-related barriers and enabling factors. Participants worked on average over 16 years in the child welfare system, suggesting that they have extensive experience working with the population. We did not assess their educational background in our assessment, which could have impacted professional views. Given that this is a nascent area of research with respect to conducting family-based telehealth interventions with families separated by the foster care system, our study was not intended to produce widely generalizable findings, which are still needed. Participants were employed in Northern and Southern California counties, suggesting that the results may be generalizable to these parts of California but may not generalize to other states or other parts of California. For example, barriers including state policies such as presumptive transfer and California Medicaid billing are state specific, while enabling factors such as transportation, foster parent involvement, and service availability are likely shared by foster youth and their families across the nation and beyond. In addition, although youth and family perspectives were discussed by the participants, they were not directly captured. The sample size of 3 focus groups with 19 participants may be viewed as limited; however, we were guided by the literature suggesting adequate saturation with relatively small sample sizes [31,32].

### Conclusions

Child welfare system professionals provided important considerations when creating and implementing a family-based intervention for foster youth and their caregivers of origin. Drawing from their perspectives, we must advocate for policies and services that are youth centered rather than placement centered, provide resources to limit barriers, bolster motivation to engage, and structure teams to promote collaboration.

## Abbreviations

CASA: court appointed special advocates
HIPAA: Health Insurance Portability and Accountability Act
STRTP: short-term residential therapeutic program

## Data Availability

The data set generated during this study is not publicly available due to ethical concerns about possible reidentification with this small study sample. Study metadata (eg, focus group guide and codebook) are available from the corresponding author on request.
